# Supplementary material for: Statistical learning occurs during practice while high-order rule learning during rest period
Source: NPJ Sci Learn. 2021 Jul 1;6:14. doi: 10.1038/s41539-021-00093-9 (PMC8249495; doi:10.1038/s41539-021-00093-9)
Supplement: Supplementary file 1 — Reporting Summary [file 41539_2021_93_MOESM1_ESM.pdf]

nature research

Corresponding author(s): Dr Romain Quentin

Last updated by author(s): 3/3/2021

Reporting Summary

Nature Research wishes to improve the reproducibility of the work that we publish. This form provides structure for consistency and transparency in reporting. For further information on Nature Research policies, see our Editorial Policies and the Editorial Policy Checklist.

Statistics

For all statistical analyses, confirm that the following items are present in the figure legend, table legend, main text, or Methods section.

n/a	Confirmed	
☐	☒	The exact sample size (*n*) for each experimental group/condition, given as a discrete number and unit of measurement
☐	☒	A statement on whether measurements were taken from distinct samples or whether the same sample was measured repeatedly
☐	☒	The statistical test(s) used AND whether they are one- or two-sided<br>*Only common tests should be described solely by name; describe more complex techniques in the Methods section.*
☐	☒	A description of all covariates tested
☐	☒	A description of any assumptions or corrections, such as tests of normality and adjustment for multiple comparisons
☐	☒	A full description of the statistical parameters including central tendency (e.g. means) or other basic estimates (e.g. regression coefficient) AND variation (e.g. standard deviation) or associated estimates of uncertainty (e.g. confidence intervals)
☐	☒	For null hypothesis testing, the test statistic (e.g. *F*, *t*, *r*) with confidence intervals, effect sizes, degrees of freedom and *P* value noted<br>*Give P values as exact values whenever suitable.*
☒	☐	For Bayesian analysis, information on the choice of priors and Markov chain Monte Carlo settings
☒	☐	For hierarchical and complex designs, identification of the appropriate level for tests and full reporting of outcomes
☐	☒	Estimates of effect sizes (e.g. Cohen's *d*, Pearson's *r*), indicating how they were calculated

Our web collection on statistics for biologists contains articles on many of the points above.

Software and code

Policy information about availability of computer code

Data collection	Eprime 2.0 was used to collect the data.
Data analysis	Analyses were done using python code. All codes can be found here: https://github.com/romquentin/Learning_during_practice_and_rest

For manuscripts utilizing custom algorithms or software that are central to the research but not yet described in published literature, software must be made available to editors and reviewers. We strongly encourage code deposition in a community repository (e.g. GitHub). See the Nature Research guidelines for submitting code & software for further information.

Data

Policy information about availability of data

All manuscripts must include a data availability statement. This statement should provide the following information, where applicable:

- Accession codes, unique identifiers, or web links for publicly available datasets
- A list of figures that have associated raw data
- A description of any restrictions on data availability

All data and code (https://github.com/romquentin/Learning_during_practice_and_rest) are available online. Further information and requests for resources should be directed to and will be fulfilled by the corresponding author, Romain Quentin (romain.quentin@inserm.fr).

Field-specific reporting

Please select the one below that is the best fit for your research. If you are not sure, read the appropriate sections before making your selection.

☐ Life sciences ☒ Behavioural & social sciences ☐ Ecological, evolutionary & environmental sciences

For a reference copy of the document with all sections, see nature.com/documents/nr-reporting-summary-flat.pdf

Behavioural & social sciences study design

All studies must disclose on these points even when the disclosure is negative.

Study description	Participants practiced a learning task typing sequence on a keyboard. Quantitative data.
Research sample	Hungarian students
Sampling strategy	Sample size was chosen based on previous studies using the same learning task.
Data collection	Data was collected on computer. Participants responded with a keyboard. A researcher was present in the room and was not aware of the hypothesis tested.
Timing	Dataset 1: 03/2008-04/2008 ; Dataset 2: 2013; Dataset 3: 10/2017-11/2017
Data exclusions	No data were excluded.
Non-participation	No participant dropped out.
Randomization	Participants were not allocated in experimental groups.

Reporting for specific materials, systems and methods

We require information from authors about some types of materials, experimental systems and methods used in many studies. Here, indicate whether each material, system or method listed is relevant to your study. If you are not sure if a list item applies to your research, read the appropriate section before selecting a response.

Materials & experimental systems

n/a	Involved in the study	
☒	☐	Antibodies
☒	☐	Eukaryotic cell lines
☒	☐	Palaeontology and archaeology
☒	☐	Animals and other organisms
☐	☐	Human research participants
☒	☐	Clinical data
☒	☐	Dual use research of concern

Methods

n/a	Involved in the study	
☒	☐	ChIP-seq
☒	☐	Flow cytometry
☒	☐	MRI-based neuroimaging

Human research participants

Policy information about studies involving human research participants

Population characteristics	268 participants (192 women, mean age = 22.2 years)
Recruitment	Participant were recruited at Hungarian universities.
Ethics oversight	United Ethical Review Committee for Research in Psychology (EPKEB) in Hungary and by the research ethics committee of Eötvös Loránd University, Budapest, Hungary

Note that full information on the approval of the study protocol must also be provided in the manuscript.
